# Pyrophosphate-Enhanced Oxidase Activity of Cerium Oxide Nanoparticles for Colorimetric Detection of Nucleic Acids

**DOI:** 10.3390/s21227567

**Published:** 2021-11-14

**Authors:** Seokhwan Kim, Jinjoo Han, Heeseok Chung, Yong-Keun Choi, Ayemeh Bagheri Hashkavayi, Yu Zhou, Ki Soo Park

**Affiliations:** 1 Department of Biological Engineering, Konkuk University, Seoul 05029, Korea; mmm1605@konkuk.ac.kr (S.K.); jinjoo9665@konkuk.ac.kr (J.H.); jhs2562@konkuk.ac.kr (H.C.); dragonrt@konkuk.ac.kr (Y.-K.C.); elnazbagheri@konkuk.ac.kr (A.B.H.); 2 College of Animal Sciences, Yangtze University, Jingzhou 434023, China

**Keywords:** cerium oxide, colorimetry, nucleic acid, oxidase activity, pyrophosphate

## Abstract

In recent years, cerium oxide (CeO_2_) nanoparticles (NPs) have drawn significant attention owing to their intrinsic enzyme mimetic properties, which make them powerful tools for biomolecular detection. In this work, we evaluated the effect of pyrophosphate (PPi) on the oxidase activity of CeO_2_ NPs. The presence of PPi was found to enhance the oxidase activity of CeO_2_ NPs, with enhanced colorimetric signals. This particular effect was then used for the colorimetric detection of target nucleic acids. Overall, the PPi-enhanced colorimetric signals of CeO_2_ NPs oxidase activity were suppressed by the presence of the target nucleic acids. Compared with previous studies using CeO_2_ NPs only, our proposed system significantly improved the signal change (ca. 200%), leading to more sensitive and reproducible colorimetric analysis of target nucleic acids. As a proof-of-concept study, the proposed system was successfully applied to the highly selective and sensitive detection of polymerase chain reaction products derived from *Klebsiella pneumoniae.* Our findings will benefit the rapid detection of nucleic acid biomarkers (e.g., pathogenic bacterial DNA or RNA) in point-of-care settings.

## 1. Introduction

Fast, robust, and ultrasensitive detection of target nucleic acids has important applications in molecular diagnostics for the detection of pathogens and viruses [1,2]. The gold standard for the detection of specific nucleic acid involves the exponential amplification of a target DNA fragment using polymerase chain reaction (PCR) followed by gel electrophoresis [3]. However, gel-based assay is not only time consuming but also requires user expertise. In recent years, real-time PCR, which can amplify DNA in real time, has been widely utilized as a promising alternative [4,5]. However, despite its high accuracy, real-time PCR requires expensive reagents (fluorescence-labeled probes or DNA binding dyes) and bulky equipment. These shortcomings become more problematic for point-of-care testing (POCT) applications [6].

In this regard, colorimetric strategies, whose results can be identified even with the naked eye, are a good option for POCT applications or facility-limited settings. Several assays are available for the rapid and sensitive colorimetric detection of target nucleic acids and other various target biomolecules [7]. The representative examples rely on metal nanomaterials (gold [8,9,10,11,12] and silver [13,14,15]) that exhibit distinct, size-dependent color changes and enzyme-mimicking activities. For example, the peroxidase-mimicking activity of magnetic nanoparticles (Fe_3_O_4_ NPs) [16] and the oxidase-mimicking activity of cerium oxide nanoparticles (CeO_2_ NPs) [17,18] catalyze the conversion of transparent substrates (e.g., 3,3′,5,5′-tetramethylbenzidine [TMB]) into colorimetric products. These catalytic reactions are suppressed by the presence of the target nucleic acids, thus leading to the development of facile colorimetric assay. These procedures are simple, and their results can be quickly analyzed without the need for expensive instruments. However, in the case of AuNPs, several factors (e.g., salt) can cause AuNPs to aggregate regardless of the presence of target nucleic acids, leading to unexpected false-positive or false-negative results [19]. In the case of Fe_3_O_4_ NPs, it requires a relatively long reaction time (ca. 30–90 min) [16,20,21] to generate the colorimetric signal and there are hydrogen peroxide (H_2_O_2_)-related toxicity issues [21,22]. Furthermore, the signal change caused by the presence of target nucleic acids is not strong enough to achieve reproducible results. On the other hand, CeO_2_ NPs with oxidase activity effectively catalyze the colorimetric reaction within a few minutes and do not involve H_2_O_2_ [23], which is more desirable for POCT application.

**Figure 1:**
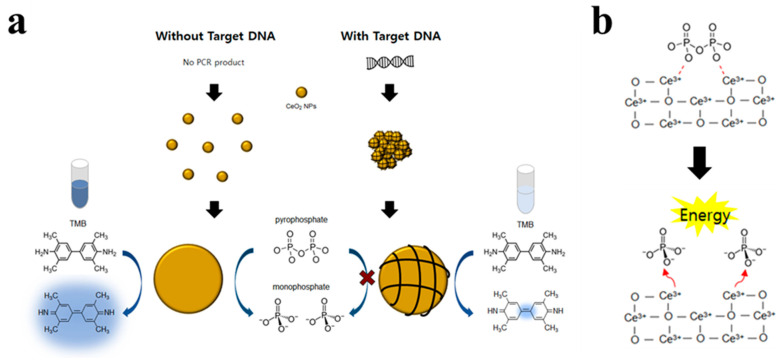
(**a**) Schematic illustration of the proposed CeO_2_ NPs-based colorimetric detection of target DNA using pyrophosphate (PPi) as an enhancer. (**b**) Reaction mechanism for the hydrolysis of phosphate ester bonds in PPi by CeO_2_ NPs.

In the current study, we aimed to devise an advanced method to enhance the colorimetric signal change caused by CeO_2_ NPs by improving their oxidase-mimicking activity. Specifically, we evaluated PPi as an effective enhancer molecule of CeO_2_ NP-catalyzed oxidation reactions in an effort to amplify the signal changes caused by the presence of target nucleic acids. We then developed the colorimetric system for the detection of target nucleic acids. As described in Figure 1a, when negatively charged nucleic acids are present, it binds to the positively charged CeO_2_ NPs through electrostatic interaction, reducing the effective surface area for the interaction with PPi and TMB substrate. As a result, the sample with target nucleic acids exhibits a suppressed colorimetric signal as compared with the one without target nucleic acids. Importantly, CeO_2_ NPs that are known to possess phosphatase-like activity as well [24,25,26] can release energy by hydrolyzing phosphate ester bonds in PPi, which thereby can contribute to the enhancement of colorimetric signal change (Figure 1b) [27]. In the effort described below, we successfully determined the target nucleic acids originated from *Klebsiella pneumoniae* with high selectivity. Results from the study may benefit the development of a molecular diagnostic system that can be used in POCT settings.

## 2. Materials and Methods

### 2.1. Reagents

Cerium (IV) oxide nanoparticles (CeO_2_ NPs) were purchased from Sigma-Aldrich (St. Louis, MO, USA). Sodium acetate, sodium phosphate, and sodium pyrophosphate were purchased from Samchun Chemical (Seoul, Korea); SYBR Green II and TMB were purchased from Thermo Fisher Scientific (Waltham, MA, USA). Deoxynucleoside triphosphate (dNTPs) and ribonucleoside triphosphate (rNTPs) were purchased from Enzynomics (Seoul, Korea). All DNA oligonucleotides were synthesized by Integrated DNA Technologies (Coralville, IA, USA). Ultrapure DNase/RNase-free distilled water from Bioneer (Daejeon, Korea) was used in all experiments. All chemicals used in this study were of analytical grade.

### 2.2. Confirmation of DNA Binding to CeO_2_ NP by Fluorescence Microscope

20 μL of 10 μM synthetic DNA (5′-AGT TCG AGCAGC AAG CTA TAT TTC CTT AAC AA-3′, 32 nt) were added to 2.7 μL of a CeO_2_ NP stock solution (2.5 wt.% colloidal dispersion in 0.4 M sodium acetate buffer), and to this solution, 22.3 μL of 0.4 M sodium acetate buffer (pH 3.7) and 41 μL of deionized water were added. After incubation for 5 min, 4 μL of 10 mM PPi was added and incubated for 30 min. Finally, images were obtained using fluorescence microscopy (KI-2000F; Korea Lab Tech, Gyeonggi-do, Korea) with filter cube (excitation: 450–480 nm; barrier: 515 nm) after adding 10 μL of 10× SYBR Green II, a staining dye specific for single-stranded DNA.

### 2.3. Bacteria Cultivation and Genomic DNA Isolation

*Klebsiella pneumoniae* (ATCC 700603), *Pseudomonas aeruginosa* (ATCC 27853), *Escherichia coli* (ATCC 25922), and *Enterobacter cloacae* (KCTC 2519) were grown in Luria-Bertani (LB) medium (BD, Franklin Lakes, NJ, USA) at 37 °C with constant shaking for 18–20 h. After the cultures were centrifuged at 5000× *g* for 5 min, the supernatant was carefully discarded, and the cell pellet was resuspended in 200 μL of the TCL buffer supplied with the Total DNA Extraction S&V Kit (Bionics, Seoul, Korea). The cells were then lysed by mixing with Proteinase K and heating for 1 h at 56 °C. Finally, genomic DNA (gDNA) was isolated according to the instructions of the gDNA extraction kit. The purity and concentration of the extracted gDNA were evaluated using a Nanodrop Spectrometer (Spectramax iD5 multi-mode microplate reader; Molecular Devices, San Jose, CA, USA) prior to storage of the gDNA at −20 °C until use.

### 2.4. PCR Amplification

Bacterial gDNA was amplified by PCR. The total reaction solution of 20 μL that contained 1 μL of bacterial gDNA, 0.5 μM of each primer, and 10 μL Topreal qRCR 2× PreMIX (SYBR Green with low ROX) (Enzynomics, Daejeon, Korea), was heat-denatured at 95 °C for 10 min, followed by 25 cycles of 95 °C for 20 s, 64 °C for 30 s, and 72 °C for 60 s. For the specific amplification of *K. pneumoniae*, *wabG* gene (GenBank accession number KX842082) was targeted, and the following primers were used for PCR amplification: forward, 5′-ACC ATC GGC CAT TTG ATA GA-3′ and reverse, 5′-CGG ACT GGC AGA TCC ATA TC-3′. After amplification, the PCR products were purified with NucleoSpin Gel & PCR Clean-up kit (Takara Bio, Kusatsu, Japan) according to the manufacturer’s protocol. The length and concentration of the PCR products were determined by agarose gel electrophoresis and Nanodrop Spectrometer (Spectramax iD5 multi-mode; Molecular Devices), respectively.

### 2.5. CeO_2_ NP-Based Colorimetric Detection Using PPi as an Enhancer

First, 20 μL of 10 μM synthetic DNA, PCR products or gDNA at different concentrations were added to 2.7 μL of a CeO_2_ NP stock solution (2.5 wt.% colloidal dispersion in 0.4 M sodium acetate buffer), and to this solution, 22.3 μL of 0.4 M sodium acetate buffer (pH 3.7) and 1 μL of deionized water were added. After incubation for 5 min, to the solution was added 50 μL of 1× TMB substrate solution and 4 μL of 10 mM PPi, which was then incubated for 30 min to develop the colorimetric signal. Not only PPi, but also other substances such as dNTP and rNTPs (4 μL, 10 mM) were tested to evaluate their enhancement effect on the CeO_2_ NP-catalyzed oxidation reactions. After centrifugation at 5900× *g* for 30 s to separate CeO_2_ NPs from the reaction solution, the colorimetric signal of the supernatant was measured at a wavelength of 650 nm using a microplate reader (Spectramax iD5 multi-mode; Molecular Devices).

## 3. Results and Discussion

### 3.1. Selection of the Best Enhancer for CeO_2_ NPs Oxidase Activity

**Figure 2:**
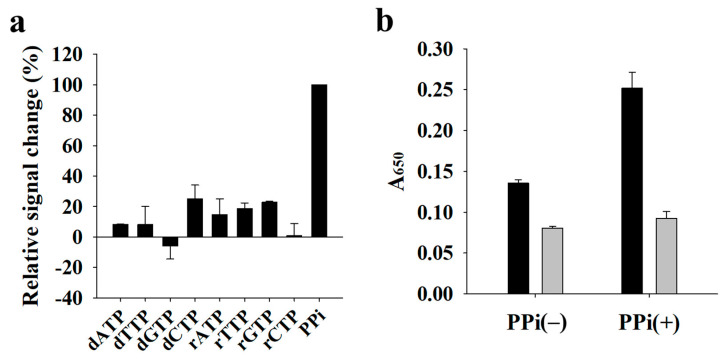
Selection of the best enhancer for CeO_2_ NPs oxidase activity. (**a**) Relative signal change (%) of dNTPs (0.4 mM), rNTPs (0.4 mM), and PPi (0.4 mM). Relative signal change (%) was calculated as the DNA-induced signal change in the presence of dNTPs and rNTPs divided by that in the presence of PPi and multiplied by 100 (%). (**b**) Absorbance signal at 650 nm (A_650_) in the absence (−) and presence (+) of PPi (0.4 mM). Black and gray bars indicate the absence and presence of synthetic DNA, respectively.

First, we investigated the effect of phosphate ester bonds using different substances, such as dNTP, rNTP, and PPi, on the oxidase activity of CeO_2_ NPs during a CeO_2_ NPs-catalyzed oxidation reaction. As shown in Figure 2a, PPi substantially increased the catalytic activity of CeO_2_ NPs and induced the highest signal change in the presence of nucleic acids. We assumed that PPi without sugar and bases can interact with the positively charged CeO_2_ NPs more effectively than dNTP and rNTP. In addition, the energy released after PPi is cleaved by CeO_2_ NPs boosted the oxidase activity of CeO_2_ NPs, resulting in substantial colorimetric signal change.

Next, using PPi as the best enhancer, we evaluated the detection feasibility of target DNA. Figure 2b shows that the presence of DNA suppressed the oxidase activity of CeO_2_ NPs, as evidenced by the low colorimetric signal at 650 nm, the maximum absorbance of oxidized TMB, regardless of the presence or absence of PPi. In contrast, the oxidase activity of CeO_2_ NPs in the absence of DNA was increased by the presence of PPi, thereby increasing the signal difference between reactions with and without DNA. Taken together, these results indicate that PPi is the key factor for the enhanced signal change in the presence of DNA.

### 3.2. Confirmation of DNA Binding to CeO_2_ NPs and Reaction Optimization

**Figure 3:**
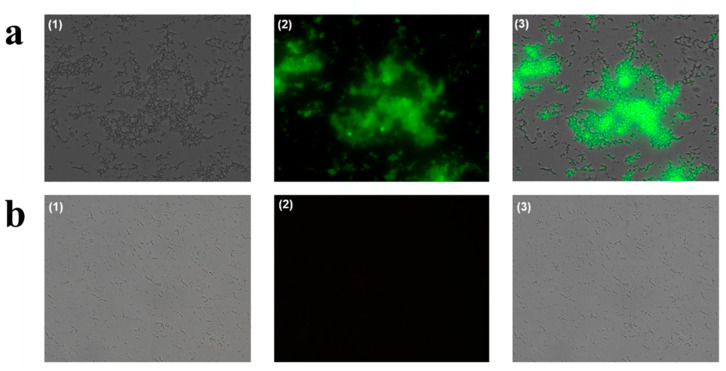
Fluorescence microscope images obtained in the presence (**a**) and absence (**b**) of DNA. (1) Optical images, (2) fluorescent images, (3) merged images.

**Figure 4:**
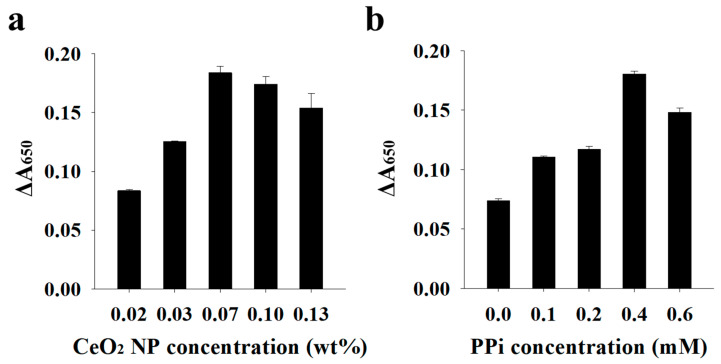
Optimization of the proposed colorimetric system. (**a**) CeO_2_ NP concentration. (**b**) PPi concentration. Change in absorbance signal (∆A_650_) was calculated by subtracting A_650_ in the presence of DNA from that in the absence of DNA.

As shown in Figure 1, the binding between the DNA and CeO_2_ NPs was assumed to drive the suppression of catalytic activity of CeO_2_ NPs. To confirm this, we investigated the adsorption of DNA onto CeO_2_ NPs using fluorescence microscopy after preparing the samples containing CeO_2_ NPs, DNA, and SYBR Green II, a staining dye specific for single-stranded DNA. Figure 3 shows that CeO_2_ NPs displayed a high fluorescence signal when the DNA was present with CeO_2_ NPs, whereas a negligible fluorescence signal was observed when the DNA was absent. This clearly confirms that the DNA binds to CeO_2_ NPs to inhibit the catalytic reaction of CeO_2_ NPs. We also optimized the reaction conditions, including the concentrations of CeO_2_ NPs and PPi, for the efficient analysis of DNA by comparing the absorbance signals in the absence and presence of DNA. Figure 4 shows that 0.07 wt.% of CeO_2_ NPs and 0.4 mM of PPi were ideal to achieve the highest signal change, which were thus used for further experiments.

### 3.3. Analytical Performance of the Proposed System

**Figure 5:**
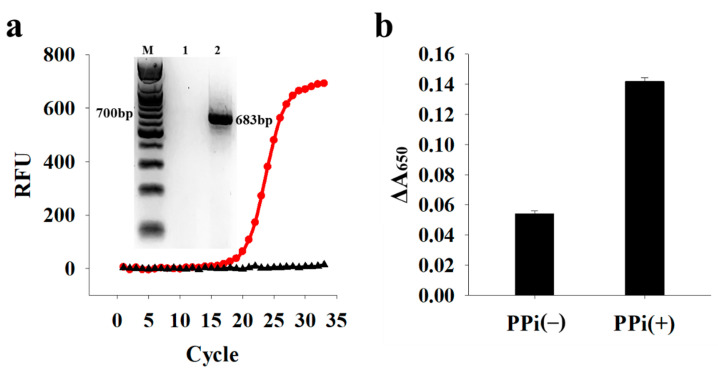
Detection of PCR products from *K. pneumoniae*. (**a**) PCR amplification curves in the absence (black) and presence (red) of gDNA from *K. pneumoniae* (1.6 × 10^3^ copies/μL). Inset shows the corresponding gel electrophoresis results. Lanes 1 and 2 indicate the samples obtained after PCR in the absence and presence of gDNA, respectively. RFU: relative fluorescence unit. (**b**) ΔA_650_ in the absence (−) and presence (+) of PPi. Change in absorbance signal (ΔA_650_) was calculated by subtracting A_650_ in the presence of target DNA from that in the absence of target DNA.

Under the optimized conditions, we demonstrated the detection feasibility of target nucleic acids originating from pathogenic bacteria. As proof of concept, we selected *K. pneumoniae* as the target pathogen and designed the specific primers by targeting *wabG* in *K. pneumoniae*. First, we verified the PCR amplification with the designed primers. As shown in Figure 5a, gDNA extracted from *K. pneumoniae* generated a PCR product with a size of 683 bp, which was distinguished from that formed in the absence of gDNA. Next, we detected the PCR product using the proposed colorimetric system, which was compared to its counterpart without using PPi. Figure 5b shows that the presence of the PCR product suppressed the colorimetric signal both without and with PPi; however, signal change (ΔA_650_) was more evident in the presence of PPi. These results were consistent with those using a synthetic target DNA (Figure 2b), demonstrating that our proposed system with PPi as enhancer is more suitable for the sensitive and selective colorimetric detection of target nucleic acids.

**Figure 6:**
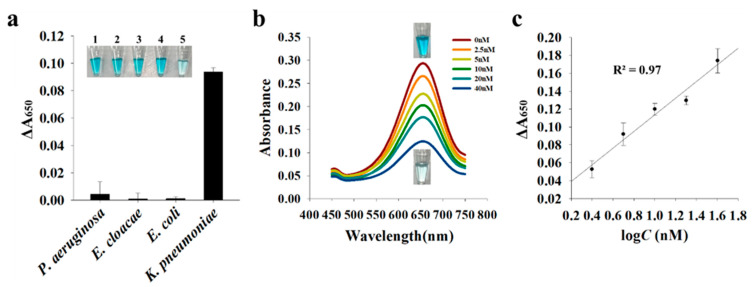
Detection selectivity (**a**) and sensitivity (**b**,**c**) of the proposed system. (**a**) Change in absorbance signal (ΔA_650_) was calculated by subtracting A_650_ in the presence of target DNA from that in the absence of target DNA. Inset shows a photographic image of each sample containing PCR products. 1: the absence of target DNA; 2: *Pseudomonas aeruginosa*; 3: *Enterobacter cloacae*; 4: *Escherichia coli*; 5: *Klebsiella pneumoniae.* (**b**) Absorbance spectra in the presence of PCR products at different concentrations and images of a control sample without PCR products and a sample containing 40 nM PCR products. (**c**) Linear relationship between ΔA_650_ and PCR concentration (2.5–40 nM).

**Figure 7:**
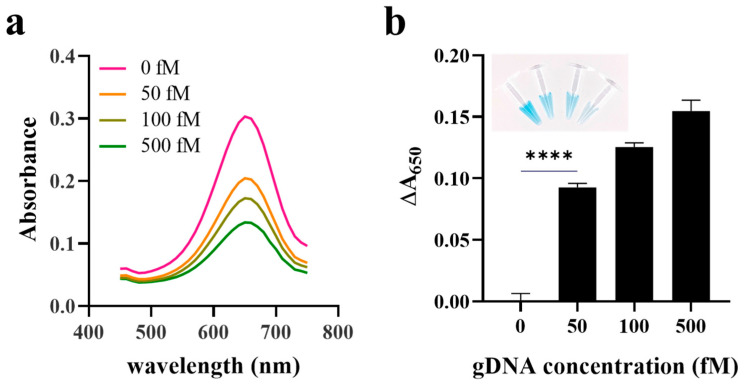
Direct detection of gDNA extracted from *Klebsiella pneumoniae*. (**a**) Absorbance spectra and (**b**) ΔA_650_ in the presence of extracted gDNA at different concentrations. Change in absorbance signal (ΔA_650_) was calculated by subtracting A_650_ in the presence of extracted gDNA from that in the absence of extracted gDNA. *p* value is indicated by stars, **** *p* < 0.0001. Inset shows a photographic image of each sample containing extracted gDNAs at different concentrations.

Next, we evaluated the selectivity and sensitivity of the proposed detection system. Because the primers were designed specifically for *K. pneumoniae*, the highest signal change (ΔA_650_) was obtained only in the presence of *K. pneumoniae*, whereas the presence of other control bacteria, including *Pseudomonas aeruginosa*, *Escherichia coli*, and *Enterobacter cloacae*, did not generate any PCR product and exhibited a negligible signal change, indicating the high specificity of the proposed system (Figure 6a). In addition, we measured the colorimetric signal in the presence of PCR products at different concentrations. Figure 6b shows that as the concentration of PCR products increased, the colorimetric signal decreased with a limit of detection (LOD) of 1.04 nM (3σ/S, where σ and S are the standard deviation of the blank and the slope). It should be noted that the LOD obtained in this assay is good enough to be used in various areas for the detection of pathogens and viruses because the general concentration of PCR products ranges from 10 to 100 nM [28]. Furthermore, the proposed system was applied to the detection of extracted gDNA from *K. pneumoniae*. The results in Figure 7 show that the presence of gDNA led to the signal change (ΔA_650_), proving the direct detection feasibility of extracted gDNA even without PCR amplification.

## 4. Conclusions

We developed a simple, colorimetric assay for the detection of target nucleic acids using the oxidase activity of CeO_2_ NPs and PPi as an enhancer to improve the oxidase activity of CeO_2_ NPs, leading to more evident colorimetric signal change. Using the proposed system, PCR products from the pathogenic bacteria, *K. pneumoniae,* were quantitatively analyzed with high selectivity. In addition, it can directly analyze gDNA extracted from target bacteria. Our findings may pave the way for the reproducible detection of various target molecules and can be used in combination with various nucleic acid amplification methods, such as isothermal nucleic acid amplification. Overall, our proposed system provides rapid colorimetric results without the need for a complicated and expensive instrument and can thus be used in POCT applications and facility-limited settings.

## Data Availability

All data generated or analyzed during this study are included in this article.

## References

[1] Lampel K.A., Orlandi P.A., Kornegay L. (2000). Improved template preparation for PCR-based assays for detection of food-borne bacterial pathogens. Appl. Environ. Microbiol..

[2] Storch G.A. (2000). Diagnostic virology. Clin. Infect. Dis..

[3] Rill R.L., Beheshti A., Van Winkle D.H. (2002). DNA electrophoresis in agarose gels: Effects of field and gel concentration on the exponential dependence of reciprocal mobility on DNA length. Electrophoresis.

[4] Espy M.J., Uhl J.R., Sloan L.M., Buckwalter S.P., Jones M.F., Vetter E.A., Yao J.D., Wengenack N.L., Rosenblatt J.E., Cockerill F.R. (2006). Real-time PCR in clinical microbiology: Applications for routine laboratory testing. Clin. Microbiol. Rev..

[5] Mackay I.M. (2004). Real-time PCR in the microbiology laboratory. Clin. Microbiol. Infect..

[6] Hauck T.S., Giri S., Gao Y., Chan W.C. (2010). Nanotechnology diagnostics for infectious diseases prevalent in developing countries. Adv. Drug Del. Rev..

[7] Cha B.S., Lee E.S., Kim S., Kim J.M., Hwang S.H., Oh S.S., Park K.S. (2020). Simple colorimetric detection of organophosphorus pesticides using naturally occurring extracellular vesicles. Microchem. J..

[8] Kalimuthu K., Cha B.S., Kim S., Park K.S. (2020). Eco-friendly synthesis and biomedical applications of gold nanoparticles: A review. Microchem. J..

[9] Hwang S.H., Jeong S., Choi H.J., Eun H., Jo M.G., Kwon W.Y., Kim S., Kim Y., Lee M., Park K.S. (2019). Target-Induced Aggregation of Gold Nanoparticles for Colorimetric Detection of Bisphenol A. J. Nanomater..

[10] Sato K., Hosokawa K., Maeda M. (2007). Colorimetric biosensors based on DNA-nanoparticle conjugates. Anal. Sci..

[11] Baptista P., Pereira E., Eaton P., Doria G., Miranda A., Gomes I., Quaresma P., Franco R. (2008). Gold nanoparticles for the development of clinical diagnosis methods. Anal. Bioanal. Chem..

[12] Li H., Rothberg L. (2004). Colorimetric detection of DNA sequences based on electrostatic interactions with unmodified gold nanoparticles. Proc. Natl. Acad. Sci. USA.

[13] Thompson D.G., Enright A., Faulds K., Smith W.E., Graham D. (2008). Ultrasensitive DNA detection using oligonucleotide–silver nanoparticle conjugates. Anal. Chem..

[14] Lee J.-S., Lytton-Jean A.K., Hurst S.J., Mirkin C.A. (2007). Silver nanoparticle− oligonucleotide conjugates based on DNA with triple cyclic disulfide moieties. Nano Lett..

[15] Xu X., Wang J., Yang F., Jiao K., Yang X. (2009). Label-free colorimetric detection of small molecules utilizing DNA oligonucleotides and silver nanoparticles. Small.

[16] Park K.S., Kim M.I., Cho D.Y., Park H.G. (2011). Label-free colorimetric detection of nucleic acids based on target-induced shielding against the peroxidase-mimicking activity of magnetic nanoparticles. Small.

[17] Kim H.Y., Ahn J.K., Kim M.I., Park K.S., Park H.G. (2019). Rapid and label-free, electrochemical DNA detection utilizing the oxidase-mimicking activity of cerium oxide nanoparticles. Electrochem. Commun..

[18] Kim M.I., Park K.S., Park H.G. (2014). Ultrafast colorimetric detection of nucleic acids based on the inhibition of the oxidase activity of cerium oxide nanoparticles. Chem. Commun..

[19] Lin L.K., Uzunoglu A., Stanciu L.A. (2018). Aminolated and Thiolated PEG-Covered Gold Nanoparticles with High Stability and Antiaggregation for Lateral Flow Detection of Bisphenol A. Small.

[20] Liu B., Liu J. (2015). Accelerating peroxidase mimicking nanozymes using DNA. Nanoscale.

[21] Liu Y., Yu F. (2011). Substrate-specific modifications on magnetic iron oxide nanoparticles as an artificial peroxidase for improving sensitivity in glucose detection. Nanotechnology.

[22] Mahaseth T., Kuzminov A. (2017). Potentiation of hydrogen peroxide toxicity: From catalase inhibition to stable DNA-iron complexes. Mutat. Res..

[23] Asati A., Santra S., Kaittanis C., Nath S., Perez J.M. (2009). Oxidase-like activity of polymer-coated cerium oxide nanoparticles. Angew. Chem. Int. Ed. Engl..

[24] Kuchma M.H., Komanski C.B., Colon J., Teblum A., Masunov A.E., Alvarado B., Babu S., Seal S., Summy J., Baker C.H. (2010). Phosphate ester hydrolysis of biologically relevant molecules by cerium oxide nanoparticles. Nanomedicine.

[25] Dhall A., Burns A., Dowding J., Das S., Seal S., Self W. (2017). Characterizing the phosphatase mimetic activity of cerium oxide nanoparticles and distinguishing its active site from that for catalase mimetic activity using anionic inhibitors. Environ. Sci. Nano.

[26] Xu C., Qu X. (2014). Cerium oxide nanoparticle: A remarkably versatile rare earth nanomaterial for biological applications. NPG Asia Mater..

[27] Ni P., Xie J., Chen C., Jiang Y., Zhao Z., Zhang Y., Lu Y., Yu J. (2019). Spectrophotometric determination of the activity of alkaline phosphatase and detection of its inhibitors by exploiting the pyrophosphate-accelerated oxidase-like activity of nanoceria. Mikrochim. Acta.

[28] Jung Y.L., Lee C.Y., Park J.H., Park K.S., Park H.G. (2018). A signal-on, colorimetric determination of deoxyribonuclease I activity utilizing the photoinduced synthesis of gold nanoparticles. Nanoscale.

